# Anlotinib: a novel multi-targeting tyrosine kinase inhibitor in clinical development

**DOI:** 10.1186/s13045-018-0664-7

**Published:** 2018-09-19

**Authors:** Guoshuang Shen, Fangchao Zheng, Dengfeng Ren, Feng Du, Qiuxia Dong, Ziyi Wang, Fuxing Zhao, Raees Ahmad, Jiuda Zhao

**Affiliations:** 1 Affiliated Hospital of Qinghai University, Affiliated Cancer Hospital of Qinghai University, Xining, 810000 China; 2 Shouguang Hospital of Traditional Chinese Medicine, Weifang, 262700 China; 3 Peking University Cancer Hospital and Institute, Beijing, 100142 China; 4 The Fifth People’s Hospital of Qinghai Province, Xining, 810000 China

**Keywords:** Anlotinib, Tyrosine kinase inhibitor, VEGFR, NSCLC, STS

## Abstract

Anlotinib is a new, orally administered tyrosine kinase inhibitor that targets vascular endothelial growth factor receptor (VEGFR), fibroblast growth factor receptor (FGFR), platelet-derived growth factor receptors (PDGFR), and c-kit. Compared to the effect of placebo, it improved both progression-free survival (PFS) and overall survival (OS) in a phase III trial in patients with advanced non-small-cell lung cancer (NSCLC), despite progression of the cancer after two lines of prior treatments. Recently, the China Food and Drug Administration (CFDA) approved single agent anlotinib as a third-line treatment for patients with advanced NSCLC. Moreover, a randomized phase IIB trial demonstrated that anlotinib significantly prolonged the median PFS in patients with advanced soft tissue sarcoma (STS). Anlotinib also showed promising efficacy in patients with advanced medullary thyroid carcinoma and metastatic renal cell carcinoma (mRCC). The tolerability profile of anlotinib is similar to that of other tyrosine kinase inhibitors that target VEGFR and other tyrosine kinase-mediated pathways; however, anlotinib has a significantly lower incidence of grade 3 or higher side effects compared to that of sunitinib. We review the rationale, clinical evidence, and future perspectives of anlotinib for the treatment of multiple cancers.

## Background

Receptor tyrosine kinases (RTKs) are transmembrane glycoproteins that communicate with cellular growth factors and extracellular ligands. They play important roles in intracellular tyrosine phosphorylation and intracellular signaling. RTK activation mediates many vital physiological processes including cell proliferation, cell growth, cell migration, cell differentiation, and apoptosis. In addition, RTKs have been implicated in a variety of pathological conditions, including cancer, metabolic and autoimmune disorders, infectious diseases, and neurodegenerative disorders.

RTK activity is regulated by protein tyrosine kinases (PTKs) and protein tyrosine phosphatases (PTPs) [1]. Normal tissues show no or low activity and expression of most oncogenic RTKs, while many malignant cells show hyperactive RTKs or upregulated oncogenic RTK levels [2, 3]. Downregulation of PTK activity can attenuate tumor cell growth, angiogenesis, and anti-apoptotic effects [4].

To date, targeted RTK inhibitors have been successfully utilized in the treatment of several cancer types [5]. Most of these inhibitors are multi-targeting drugs such as imatinib, sorafenib, sunitinib, and pazopanib, which achieve therapeutic efficacy in some tumors. For example, sorafenib inhibits multiple targets including the vascular endothelial growth factor (VEGF) receptors, VEGFR-1, VEGFR-2, and VEGFR-3, as well as Raf serine/threonine kinases and platelet-derived growth factor receptor (PDGFR)-β [5, 6]. Sunitinib can inhibit VEGFR types 1 and 2 (i.e., FLT1 and FLK1/KDR, respectively), PDGFR-α, PDGFR-β, the stem cell factor receptor c-KIT, as well as FLT3 and RET kinases [7]. In patients with renal cell carcinoma (RCC), sorafenib can significantly improve progression-free survival (PFS) from 2.8 to 5.6 months compared to that of placebo [8] and sunitinib can increase PFS from 5.0 to 11.0 months compared to that of interferon (IFN) [9]. Regorafenib can inhibit the activity of both angiogenic (VEGFR1, VEGFR2, VEGFR3, TIE2), stromal (PDGFR, FGFR), and oncogenic (KIT, RET, RAF-1, BRAF, BRAF^V600E^) receptor tyrosine kinases, as well as the activity of Abl. It also significantly prolongs both overall survival (OS) and PFS in patients with refractory metastatic colorectal cancer [10, 11]. Pazopanib targets several RTKs, including VEGFR-1, VEGFR-2, VEGFR-3, and PDGFR-α. Compared with that of placebo, pazopanib showed significant prolongation of PFS in patients with advanced nonadipocytic soft tissue sarcoma (STS) (1.6 months versus 4.6 months) [12].

Nevertheless, considering the unsatisfactory efficacies and limitations of current therapies for the different stages of many cancers, there is still a need to develop innovative, more effective, and safer anticancer drugs. For example, there is currently no standard third-line treatment for advanced non-small-cell lung cancer (NSCLC). Moreover, although several targeted drugs, including olaratumab, pazopanib, sunitinib, and everolimus, are efficacious for STS according to the National Comprehensive Cancer Network (NCCN) and European guidelines [13, 14], the current targeted-therapy drugs for non-gastrointestinal stromal tissue (GIST) STS are still limited. Indeed, pazopanib is the only small-molecule tyrosine kinase inhibitor (TKI) approved by the Food and Drug Administration (FDA) as a second-line non-GIST STS treatment. In addition, no standard treatment is available to date in China for patients with STS who progressed after first-line chemotherapy [15]. For these reasons, multi-targeting RTK inhibitors are one of the most popular and important drug classes being studied, and they may play a significant role in the treatment of cancers.

## Anlotinib: a novel inhibitor that targets multiple RTKs

**Fig. 1:**
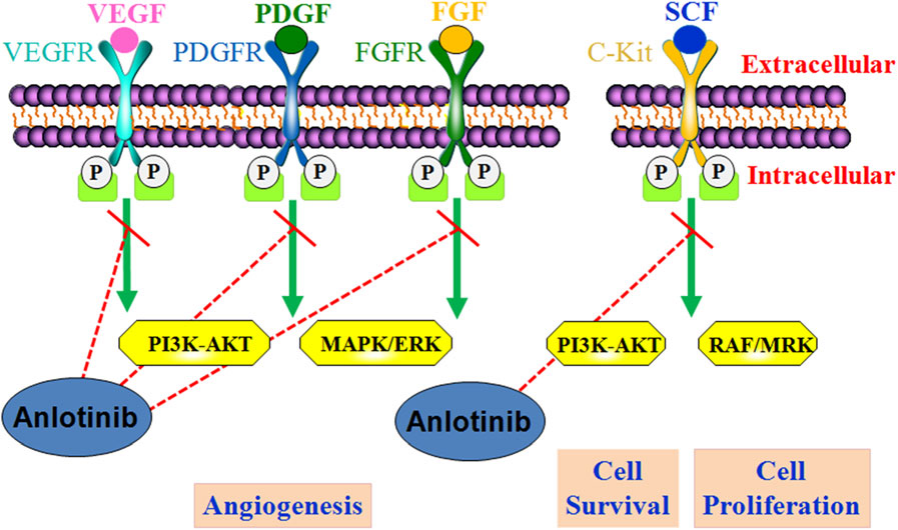
Mechanism of action of anlotinib

Anlotinib (1-[[4-(4-fluoro-2-methyl-1H-indol-5-yloxy)-6- methoxyquinolin-7-yl]oxy] methyl]cyclopropanamine dihydrochloride) is a newly developed oral small-molecule RTK inhibitor that targets VEGFR1, VEGFR2/KDR, VEGFR3, c-Kit, PDGFR-α, and the fibroblast growth factor receptors (FGFR1, FGFR2, and FGFR3). Further, it can inhibit both tumor angiogenesis and tumor cell proliferation [16, 17] (Fig. 1). Anlotinib can inhibit more targets than other RTK inhibitors can, including sorafenib, sunitinib, and pazopanib. The various targets of anlotinib and other RTK inhibitors are summarized in Table 1. Anlotinib was developed by Chia-tai Tianqing Pharmaceutical Co., Ltd. in China.

**Table 1:** The different targets between anlotinib and other RTK inhibitors

	VEGFR			PDGFR		FGFR				Others
	1	2	3	α	β	1	2	3	4	
Sorafenib	+	+	+	–	+	–	–	–	–	RET (+), c-KIT(+), FLT3(+)
Sunitinib	+	+	+	+	+	–	–	–	–	FLT3(+), c-KIT(+), RET(+), CSF1R(+)
Axitinib	+	+	+	–	–	–	–	–	–	–
Vatalanib	+	+	+	–	+	–	–	–	–	c-KIT(+)
Nintedanib	+	+	+	+	+	+	+	+	–	FLT3(+), Src(+)
Pazopanib	+	+	+	+	+	+	–	–	–	c-KIT(+)
Anlotinib	+	+	+	+	+	+	+	+	+	c-KIT(+)

+ = target, − = no target

Preclinical studies have shown that anlotinib inhibits cell migration and the formation of capillary-like tubes induced by VEGF/PDGF-BB/FGF-2 in endothelial cells. Furthermore, anlotinib significantly suppressed VEGF/PDGF-BB/FGF-2-induced angiogenesis in vitro and in vivo. Research into possible mechanisms indicated that anlotinib inhibits the activation of VEGFR2, PDGFRβ, and FGFR1, as well as downstream ERK signaling. The anti-angiogenic activity of anlotinib is stronger than that of three other anti-angiogenesis drugs, including sunitinib, sorafenib, and nintedanib [18]. Another study revealed that anlotinib binds to the ATP-binding pocket of VEGFR2 tyrosine kinase and inhibits VEGFR2 with high selectivity (IC_50_ < 1 nmol/L), thereby inhibiting VEGF-stimulated proliferation of human umbilical vein endothelial cells (HUVECs). Moreover, anlotinib suppressed HUVEC migration, tube formation, and microvessel growth in vitro and reduced vascular density in vivo. Anlotinib had broader and better antitumor efficacy than did sunitinib in vivo [16]. In cell lines expressing mutated FGFR2 protein, anlotinib decreased the number of cells. Nevertheless, similar to that of other oral RTK inhibitors, the combined treatment of anlotinib with carboplatin and paclitaxel did not appear to be more efficacious than anlotinib alone [19].

## Anlotinib dosing and pharmacokinetics

The pharmacokinetic properties of anlotinib have been estimated in studies on animals and patients with advanced solid tumors [20–22].

The results of pharmacokinetic and disposition investigations in rats and dogs showed that anlotinib had good membrane permeability and was absorbed quickly. The oral bioavailability of anlotinib was 28–58% and 41–77% in rats and dogs, respectively. The biotransformation of anlotinib showed a significant difference between species, with a terminal half-life of 22.8 ± 11.0 h in dogs and 5.1 ± 1.6 h in rats. The difference appeared to be related to differences in total plasma clearance (rats, 5.35 ± 1.31 L/h/kg; dogs, 0.40 ± 0.06 L/h/kg). Anlotinib had large apparent volumes of distribution in rats (27.6 ± 3.1 L/kg) and dogs (6.6 ± 2.5 L/kg). It was highly bound to plasma in all species, including rat (97%), dog (96%), and human (93%). In human plasma, anlotinib was bound mainly to albumin and lipoproteins. The levels of anlotinib in the tissues of rats and tumor-bearing mice were significantly higher than the corresponding level in the plasma [20].

In vitro, anlotinib could be metabolized by various human cytochrome P450 isoforms; CYP3A4 and CYP3A5 were mainly responsible. This suggests that circulating anlotinib levels are easily influenced by hepatic drugs that alter the function of P450 enzymes [20]. In vivo, anlotinib exerted a significant effect on the induction of CYP2D1 and CYP3A1/2, while it did not have a significant effect on CYP1A2, CYP2D2, or CYP2C6 after oral administration in rats. Caution, therefore, is warranted when anlotinib is administered with other drugs that are metabolized by CYP2D1 and CYP3A1/2 [23].

There have been two phase I clinical trials investigating the pharmacokinetic properties of anlotinib. In a phase I clinical study in China, the plasma concentration of anlotinib increased significantly 1 h after dosing in most patients, confirming that anlotinib was rapidly absorbed from the intestines. The peak plasma concentration (*C*_max_) and area under the concentration-time curve to 120 h post-dose (AUC_0–120 h_) of anlotinib both increased with increasing doses from 5 to 16 mg anlotinib/person, while the dose proportionality was indeterminate [21]. After a dose of 16 mg anlotinib/person, the mean *C*_max_ of anlotinib was 10.5 ± 2.9 ng/mL. The time taken to achieve *C*_max_ (*T*_max_) and the elimination half-life (*t*_1/2_) of anlotinib were 4–11 h and 96 ± 17 h following dosing, respectively [21]. Anlotinib has a significantly longer *t*_1/2_ in patients than do most tyrosine kinase inhibitors that have been used clinically to date (i.e., 3–60 h) [24]. This extremely long *t*_1/2_ leads to a significant accumulation of plasma anlotinib over time, with a mean accumulation ratio (Rac) of 12 ± 7. A 2-week subchronic dosing regimen resulted in the continuous elevation of plasma anlotinib concentration, with the maximum achieved on day 14. Thereafter, the plasma level of anlotinib decreased over a 7-day washout period. Based on the above data and toxicity profile (see toxicity section below), this phase I study recommended a dosing regimen in future studies of 12 mg daily for 2 weeks, followed by a 1-week break [21].

In another phase I study performed in a Caucasian population, the average *T*_max_ and *C*_max_ after a single 12-mg dose of anlotinib were 10 (4–24) h and 9.60 (8.47–11.50) ng/mL, respectively, which were very similar to the values found in the Chinese patients. Following multiple, continuous doses of anlotinib, the average *T*_max_ and *C*_max_ were 360 h and 63.80 (52.90–80.30) ng/mL, respectively. The authors of this phase I study also suggested a dosing regimen of 12 mg daily, 2 weeks on/1 week off for phase II studies [22].

## Therapeutic efficacy of anlotinib

### Advanced NSCLC

NSCLC is one of the most common diseases used to study the efficacy and safety of anlotinib. As no standard third-line treatment is available for patients with advanced NSCLC, anlotinib has mainly been investigated in this population to date.

A randomized, double-blind, multicenter, placebo-controlled phase II clinical trial was conducted to estimate the safety and efficacy of anlotinib monotherapy for refractory NSCLC in patients who had failed at least two types of systemic chemotherapy or experienced drug intolerance, revealing the roles of anlotinib as a third-line therapy or beyond in previously treated NSCLC [25]. Overall, 117 patients were enrolled and randomized 1:1 to receive anlotinib (12 mg per day, per os; days 1–14; 21 days per cycle) or placebo. Patients receiving anlotinib had longer PFS than patients receiving placebo (4.8 vs 1.2 months; hazard ratio (HR) = 0.32; 95% confidence interval (CI), 0.20–0.51; *P* < 0.0001). Moreover, the overall response rate (ORR) in the anlotinib group was greater than that in the placebo group (10.0%; 95% CI 2.4–17.6% vs 0%; 95% CI 0–6.27%; *P* = 0.028). Notably, anlotinib treatment benefited all subgroups independent of age, sex, smoking history, stage, efficacy of previous treatments, and histology, except the subgroup with three or fewer metastases. In addition, the OS was longer in the anlotinib group than in the control group, although the difference was not statistically significant (9.3 vs 6.3 months; HR = 0.32; 95% CI 0.51–1.18; *P* = 0.2316). The failure in obtaining a statistically significant difference in the OS might be related to the small sample size.

ALTER-0303 was a randomized, double-blind, placebo-controlled, multicenter, phase III trial that compared the efficacy and safety of anlotinib with that of placebo in patients with advanced NSCLC who progressed after at least two lines of prior treatments [26]. A total of 437 patients were randomized 2:1 to receive either oral anlotinib or placebo (12 mg QD from days 1 to 14 of a 21-day cycle). Treatment continued until tumor progression or discontinuation due to toxicity. Patients with epidermal growth factor receptor (EGFR) mutations or anaplastic lymphoma kinase (ALK) translocations who were enrolled in the study must have had treatment failure with prior targeted therapies. The results showed that anlotinib was more effective than placebo in third-line treatment of patients with advanced NSCLC. Improvements in the ORR and disease control rate (DCR) were seen in the anlotinib group compared with that of the placebo group (ORR 9.18% vs 0.7%, *P* < 0.0001; DCR 80.95% vs 37.06%, *P* < 0.0001). In addition, anlotinib significantly prolonged median PFS and OS compared with the placebo values (PFS 5.37 vs 1.40 months: HR = 0.28, 95% CI 0.19–0.31, *P* < 0.0001; OS 9.63 vs 6.30 months: HR = 0.68, 95% CI 0.54–0.87, *P* < 0.0001).

An exploratory subgroup analysis of the ALTER0303 trial showed that anlotinib significantly improved PFS and OS in patients with both sensitive EGFR mutations and wild-type EGFR. The PFS and OS in patients with sensitive EGFR mutations receiving anlotinib and placebo were 5.57 months and 0.83 months (PFS, HR = 0.15, 95% CI 0.09–0.24, *P* < 0.0001), respectively, and 10.70 months and 6.27 months (OS, HR = 0.59, 95% CI 0.37–0.93, *P* = 0.0227), respectively. Furthermore, the PFS and OS in patients with wild-type EGFR receiving anlotinib and placebo were 5.37 months and 1.57 months (PFS, HR = 0.29, 95% CI 0.22–0.39, *P* < 0.0001), respectively, and 8.87 months and 6.47 months (OS, HR = 0.73, 95% CI 0.55–0.97, *P* = 0.0282), respectively [27]. More recently, the study investigators reported that anlotinib led to a greater improvement in OS time in patients with sensitive EGFR mutations than in those with wild-type EGFR (10.70 vs 8.87, HR = 0.685, 95% CI 0.50–0.95, *P* = 0.0204) [28]. Other studies have shown that anlotinib increases survival in patients with adenocarcinomas or squamous cell carcinomas [29] and in elderly patients (over 70 years) [30]. The PFS and OS benefit from anlotinib was also independent of any previous therapeutic strategy, including conventional platinum-based chemotherapy or TKIs (gefitinib, erlotinib, and icotinib) [31].

Based on the results of ALTER-0303, anlotinib was approved by the China Food and Drug Administration (CFDA) for third-line treatment or beyond in advanced NSCLC on May 8, 2018, in China [32]. Moreover, anlotinib is recommended in the Chinese Society of Clinical Oncology Guidelines for the Diagnosis and Treatment of Primary Lung Cancer (2018 Edition) for the same indication [33].

### Advanced STS

In recent years, an increasing number of targeted drugs have demonstrated good clinical efficacy in patients with certain histological types of advanced STS. These agents include multi-targeted kinase inhibitors, such as pazopanib, imatinib, sunitinib, and sorafenib; ALK inhibitors, such as crizotinib and ceritinib; anti-PDGFRs, such as the anti-PDGFRα monoclonal antibody olaratumab; and anti-angiogenic drugs, such as bevacizumab [12, 34–41]. However, pazopanib is the only small molecule TKI approved by the FDA for second-line STS treatment to date.

Considering that anlotinib induced tumors to shrink in soft tissue sarcomas in a phase I study, a multicenter, single-arm, phase II study subsequently explored anlotinib activity in patients with advanced STS who had failed previous conventional treatments [42]. The enrolled patients had malignant fibrous histiocytoma (MFH), liposarcoma, leiomyosarcoma (LMS), synovial sarcoma (SS), or other sarcomas, but not rhabdomyosarcoma (RMS), chondrosarcoma, or GIST STS. Among the 166 patients included, the progression-free rate at week 12 (PFR_12w_) was 57.23%, median PFS was 5.63 months, and the ORR was 11.45%. Overall, anlotinib demonstrated better clinical benefits in many pathological types of STS. Specifically, alveolar soft part sarcoma (ASPS) showed a high PFR_12w_ (76.92%), similar to the efficacy of sunitinib toward ASPS [43].

The study team further conducted a phase IIB study to demonstrate the role of anlotinib in advanced STS. Overall, 233 patients who were treatment-intolerant or progressed on anthracycline-based chemotherapy were enrolled. The included pathological subtypes were SS, ASPS, LMS, and others; participants with each type were randomized 2:1 to receive anlotinib or placebo. The ORR and DCR in the anlotinib group were significantly higher than those in the control group (ORR 10.13% vs 1.33%, *P* = 0.0145; DCR 55.7% vs 22.67%, *P* < 0.0001). Additionally, anlotinib treatment significantly improved the median PFS relative to the control (6.27 months, 95% CI 4.30–8.40 vs 1.47 months, 95% CI 1.43–1.57, HR = 0.33, *P* < 0.0001). The pathological subtype with the greatest increase in survival was ASPS, whose median PFS was 18.23 months in the anlotinib group compared with 3 months in the control group (HR = 0.14, *P* < 0.0001). This trial further confirmed the efficacy and safety of anlotinib in advanced STS [44].

The results of these two clinical studies were presented in the oral report section at the American Society of Clinical Oncology (ASCO) annual meeting due to the excellent therapeutic efficacy of anlotinib in STS. It is likely that anlotinib will be approved to treat STS in China in the future.

### Metastatic renal cell carcinoma

More recently, a number of targeted treatments have become widely used as first- and second-line treatments in patients with metastatic renal cell carcinoma (mRCC). Multi-targeting kinase inhibitors, including pazopanib, sunitinib, sorafenib, cabozantinib, axitinib, and lenvatinib, were all efficacious in these patients [8, 9, 45–49].

Two phase II clinical trials have also assessed anlotinib efficacy in the treatment of mRCC. Sequential treatment with targeted therapies is effective and has been the current standard of care for patients with mRCC who failed a previous therapy. A multicenter, single-arm, phase II trial enrolled 43 patients who progressed while on, or were intolerant to, sorafenib or sunitinib. The median PFS in the whole group and in patients who had progressed while being treated with a TKI was 11.8 and 8.5 months, respectively. In intention-to-treat (ITT) patients, the ORR and 6-week DCR were 19.1% (95% CI 8.60–34.12) and 90.5% (95% CI 77.4–97.3%), respectively. Anlotinib was preliminarily shown to have promising efficacy with a favorable toxicity profile for patients with mRCC who failed sorafenib or sunitinib treatment [50].

The same authors also conducted a multicenter randomized phase II trial to compare the efficacies and safeties of anlotinib and sunitinib as first-line treatments in patients with mRCC. One-hundred and thirty-three patients (93 with anlotinib, 40 with sunitinib) were enrolled. The results showed that the anlotinib and sunitinib groups had similar PFS (11.3 vs 11.0 months, *P* = 0.30), ORR (24.4% vs 23.3%), and 6-week DCR (97.8% vs 93.0%, *P* = 0.33). More importantly, the incidence of over-grade 3 side effects was lower in the anlotinib group than in the sunitinib group (28.9% vs 55.8%, *P* = 0.0039), particularly for grade 3 or 4 thrombocytopenia (0 vs 11.6%, *P* = 0.003) and neutropenia (0.0 vs 9.3%, *P* = 0.009). These results support the hypothesis that anlotinib has a similar efficacy, but milder side effects, to that of sunitinib in patients with mRCC [51].

### Advanced medullary thyroid cancer

The kinases RET and VEGFR2 are the main targets of agents used in patients with advanced medullary thyroid cancer (MTC). Several multi-targeting kinase inhibitors, such as sorafenib, sunitinib, cabozantinib, vandetanib, and pazopanib, have shown promise in patients with differentiated and advanced MTC [52–56].

A single-arm, multicenter phase II trial estimated the efficacy and safety of anlotinib in advanced MTC. The trial enrolled 58 patients with advanced or relapsed MTC, who could not receive radical surgery, and treated them with anlotinib. The average PFS was 12.8 months (median PFS not reached), the overall ORR was 48.28% (full analysis set, FAS), and the DCR at weeks 24 and 48 was 92.16% and 84.53%, respectively. These results indicate that anlotinib has the potential to treat advanced MTC [57].

The existing clinical trial treatment efficiency data of anlotinib are summarized in Table 2. In addition, a phase III trial of anlotinib in treating metastatic colorectal cancer is completed and the results will be released in the near future.

**Table 2:** Summary of clinical efficacy results evaluating anlotinib in patients with cancer

Cancer type	Phase	Number of patients	ORR, % (anlotinib group).	ORR, % (control group).	PFS (median, months, anlotinib group).	PFS (median, months, control group).	OS (median, months, anlotinib group).	OS (median, months, control group).	Author	Ref
Advanced NSCLC	II (randomized control)	117	10.0	0.00	4.8	1.2	9.30	6.30	Han B	[25]
Advanced NSCLC	III	437	9.18	0.7	5.37	1.40	9.63	6.30	Han B	[26]
Advanced STS	II (single-arm)	166	11.45	/	5.63	/	NA	/	Chi Y	[42]
Advanced STS	IIB (randomized control)	233	10.13	1.33	6.27	1.47	NA	NA	Chi Y	[44]
mRCC	II (single-arm)	43	19.1	/	11.8 (whole group);8.5(progressed on a TKI group)	/	NA	NA	Zhou A P	[50]
mRCC	II (randomized control)	133	24.4	23.3	11.3	11.0	NA	NA	Zhou AP	[51]
Advanced MTC	II (single-arm)	58	48.28	/	12.8	/	NA	/	Sun Y	[57]

*NSCLC* non-small-cell lung cancer, *ORR* overall response rate, *PFS* progression-free survival, *OS* overall survival, *NA* data not available, *STS* soft tissues sarcoma, *mRCC* metastatic renal cell carcinoma, *TKI* tyrosine kinase inhibitor, *MTC* medullary thyroid cancer

## Anlotinib tolerability

Once-daily anlotinib 12 mg, administered as 2 weeks on/1 week off, was the suggested regimen from a phase I study. This dosage and administration schedule was used in all subsequent phase II–III trials.

**Fig. 2:**
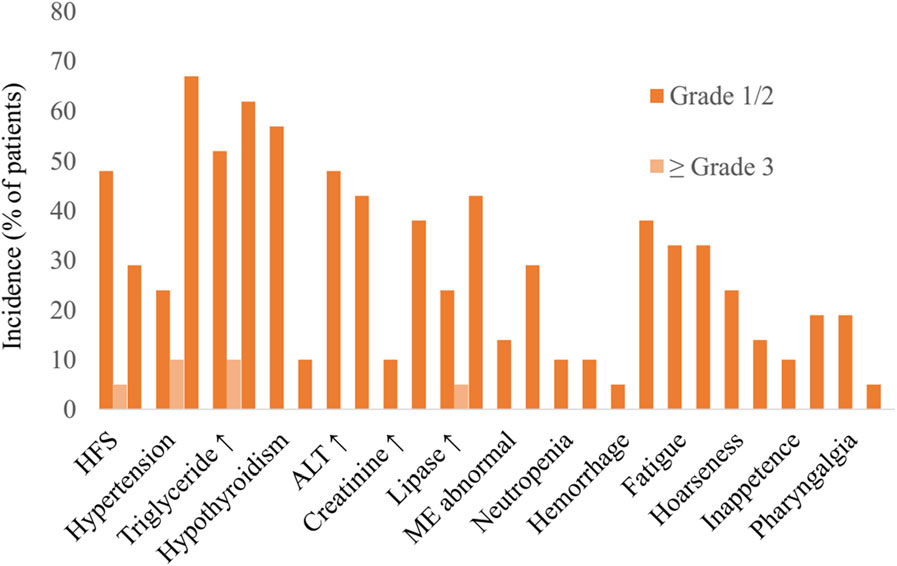
Treatment-related adverse events associated with 2-week on/1-week off anlotinib 12 mg/day. Adverse events reported in all patients (*n* = 21) in a phase I trial [21]. *HFS* hand-foot skin reaction, *ALT* alanine aminotransferase, *ME* myocardial enzymes

All adverse events (AEs) appeared to be manageable in the phase I trial. The most common AEs with over 30% incidence were hand-foot skin reaction (53%), hypertension (34%), proteinuria (67%), triglyceride elevation (62%), total cholesterol elevation (62%), hypothyroidism (57%), alanine aminotransferase (ALT) elevation (48%), aspartate transaminase (AST) elevation (43%), total bilirubin elevation (38%), serum amylase (43%), myocardial enzymes abnormal (38%), leucopenia (33%), and neutropenia (33%) [21]. The overall incidence of any AE with anlotinib was 100%, while 29% of patients reported grade 3/4 AEs, including hand-foot skin reaction (5%), hypertension (10%), triglyceride elevation (10%), and lipase elevation (5%) (Fig. 2) [21]. As the authors indicated, it is noteworthy that anlotinib appeared to cause less and milder diarrhea than did other oral anti-VEGFR TKIs [58–60]. However, it also should be noted that patients receiving anlotinib treatment had a high occurrence of triglyceride and cholesterol elevation. Although these effects did not induce noticeable symptoms, the authors suggested that patients taking anlotinib undergo regular monitoring, particularly considering that some of the listed AEs are related to arterial thromboembolic events; such events were significantly more common in patients treated with anti-VEGFR TKIs, however [61].

Anlotinib had a similar toxicity in another phase I trial and a manageable AE profile in phase II–III trials. Among 58 patients with advanced MTC who received anlotinib treatment in a phase II study, 20.7% required a dose adjustment to 10 mg daily in a 2 weeks on/1 week off schedule because of grade III/IV AEs [57]. It is noteworthy that 5 of 166 patients (3.01%) with advanced STS treated with anlotinib experienced grade III/IV pneumothorax [42]. Additionally, in patients with mRCC, anlotinib induced significantly fewer cases of grade 3/4 side effects, especially thrombocytopenia and neutropenia, than sunitinib did, but caused a greater incidence of hypercholesterolemia [51]. In a phase III trial, grade 3 or higher AEs, including dermal toxicity (3.74%) and hypertriglyceridemia (3.06%), were reported in patients with refractory advanced NSCLC administered anlotinib as a third-line treatment. However, there were no treatment-related deaths [26]. More recently in a phase II trial, additional grade 3 or higher AEs, including hyponatremia (3.16%) and neutrophil count reduction (3.16%), were observed in patients with metastatic STS receiving anlotinib treatment [44].

## Biomarkers

Appropriate biomarkers can accurately predict and monitor early efficacy and indicate emerging resistance to anlotinib. Fortunately, several clinical trials have identified circulating biomarkers that predict anlotinib activity, specifically activated circulating endothelial cells (aCECs) and EGFR-sensitizing mutations or T790M mutation.

In the ALTER0303 trial, aCECs were measured in 49 patients receiving anlotinib and 30 patients receiving placebo. There were no statistically significant differences in baseline characteristics between the groups. Using a cutoff of 1 for the ratio of the minimal aCEC numbers at every time point to baseline (aCEC min/baseline), the 49 patients receiving anlotinib were subdivided into two groups. The median PFS of the aCEC min/baseline < 1 group (35 patients) was longer than that of the aCEC min/baseline > 1 group (14 patients) (193 vs 124 days, HR = 0.439, 95% CI 0.211–0.912, *P* = 0.023). However, there was no significant relationship between PFS and the number of aCEC min/baseline in patients receiving placebo. Therefore, aCECs are a potential biomarker for PFS during anlotinib treatment [62].

The ALTER0303 trial also estimated whether circulating tumor DNA (ctDNA) levels can predict the efficacy of anlotinib treatment in patients with advanced NSCLC. Overall, 92 blood samples were analyzed through capture-based targeted ultradeep sequencing. The results revealed that 58% (53/92) had driver mutations. The maximum mutation allele frequency (MAF) at baseline had a reciprocal effect on PFS (HR = 0.612, 95% CI 0.402–0.932, *P* = 0.006). Moreover, there was no correlation between sensitizing EGFR mutations and PFS in 27 patients (5.53 vs 5.53 months, HR = 1.16, 95% CI 0.73–1.85, *P* = 0.495). Similarly, the EGFR T790M mutation did not reflect the treatment effectiveness of anlotinib in 17 patients with advanced NSCLC (5.53 vs 5.53 months, HR = 1.35, 95% CI 0.75–2.41, *P* = 0.253). Considering the possibility of bias found in small samples, an ongoing, larger scale analysis is needed to reaffirm these conclusions [63].

## Ongoing clinical trials

Clinical trials have been conducted to estimate the efficacy and side effects of anlotinib in several advanced solid tumors, including sarcomas, hepatocellular carcinoma, thyroid carcinoma, esophageal squamous cell carcinoma, gastroenteropancreatic neuroendocrine tumor G3, colorectal cancer, gastric cancer, mRCC, small cell lung cancer, and NSCLC (Table 3).

**Table 3:** Current anlotinib clinical trials for multiple cancers

Regimen	Study type	Enrollment	Population
Anlotinib and irinotecan	Phase III	Recruiting	Advanced Ewing sarcoma
Anlotinib	Phase II/III	Not recruiting	Advanced soft tissue sarcoma
Anlotinib	Phase II	Unknown status	Soft tissue sarcoma
Anlotinib	Phase III	Recruiting	Metastatic or advanced alveolar soft part sarcoma, leiomyosarcoma and synovial sarcoma
Anlotinib	Phase II	Recruiting	Hepatocellular carcinoma
Anlotinib	Phase II/III	Recruiting	Medullary thyroid carcinoma
Anlotinib	Phase II/III	Recruiting	Differentiated thyroid cancer
Anlotinib	Phase II	Not recruiting	Advanced renal cell carcinoma
Anlotinib	Phase II	Recruiting	Esophageal squamous cell carcinoma
Anlotinib plus irinotecan	Phase II	Not recruiting	Esophageal squamous cell carcinoma
Anlotinib	Phase II	Recruiting	Gastroenteropancreatic neuroendocrine tumor G3
Anlotinib	Phase II	Not recruiting	Colorectal cancer
Anlotinib	Phase II	Recruiting	Small cell lung cancer
Anlotinib	Phase II/III	Recruiting	Gastric cancer
Anti-angiogenesis plus EGFR-TKI	Phase II	Not recruiting	Non-squamous non-small cell lung cancer

*EGFR* epidermal growth factor receptor, *TKI* tyrosine kinase inhibitor

Several ongoing trials are attempting to clarify the roles of anlotinib in STS, particularly the activity of anlotinib in several STS subtypes, such as Ewing sarcoma, ASPS, leiomyosarcoma, and synovial sarcoma. It is noteworthy that most of these are phase III studies due to the rare incidence of STS. Further, several trials are attempting to determine the efficacy of anlotinib in gastrointestinal tumors. Considering the very limited number of multi-targeting RTK inhibitors that have shown efficacy in esophageal squamous cell carcinoma and gastric cancer to date, it is important to fully understand the potential role of anlotinib treatment in these tumors. Moreover, phase I trials in neuroendocrine cancer, for which there are no ideal targeted drugs, have shown evidence of anlotinib treatment efficacy; clinical trials have also been designed to evaluate the efficacy of anlotinib treatment in gastroenteropancreatic neuroendocrine tumor G3 and small cell lung cancers.

## Future perspectives

Anlotinib has exceptional efficacy and acceptable toxicity for the treatment of advanced NSCLC and STS. Anlotinib received its first approval on May 8, 2018, for patients with advanced NSCLC who have progressed after at least two lines of prior treatments in China. In the near future, it is very likely that anlotinib will also be approved in China to treat patients with STS who failed previous conventional treatments. Anlotinib also has potential as a new treatment for other solid tumors, such as mRCC and thyroid carcinoma.

Although anlotinib showed activity against several cancers, there are still some questions that require further investigation and solutions prior to its general use. First, predictive biomarkers should be further investigated to help select optimal patients for anlotinib treatment. Although some biomarkers appeared to define patients who are most likely to benefit from anlotinib treatment, the overall number of predictive biomarkers is insufficient. Moreover, the current potential biomarkers were identified from one clinical trial that used a limited number of samples from NSCLC patients. Predictive biomarkers for other cancer types remain elusive. Future trials should further investigate the optimal indications for patients likely to benefit from anlotinib.

Second, an estimate of the efficacy of anlotinib in other tumors and the appropriate schedule of anlotinib when combined with other treatments is still needed. Whether anlotinib can be expanded to the treatment of other cancers or as a first-line drug, particularly in some subtypes of STS, also requires further study. Generally, multi-targeting RTK inhibitors, such as sunitinib, sorafenib, and regorafenib, can be used to treat multiple cancers. As a novel agent, anlotinib is assumed to be effective only in limited cancer types while studies on other tumors are still ongoing. It is expected that anlotinib will show efficacy against other tumors. Therefore, more high-quality, randomized trials should be conducted to define its therapeutic efficacy in other diseases. Additionally, it is likely that anlotinib will perform different treatment roles in different cancer types. Thus, the optimal anlotinib treatment regimen for these cancers needs further evaluation.

Moreover, when the anti-angiogenesis drug ramucirumab is combined with chemotherapy, a synergistic effect is seen [64, 65]. Indeed, some targeted therapies can also modulate the host immune response and, therefore, may further improve clinical outcomes when combined with immunotherapies. Nevertheless, most studies to date have only examined anlotinib monotherapy treatments [66, 67]. Thus, future studies are needed to evaluate the combination of anlotinib and other therapies. Considering the strong efficacy of anlotinib in ASPS, further study is needed to estimate whether anlotinib could be administered as a first-line treatment in these patients.

Third, the long-term toxicity profile of anlotinib remains unclear and requires further study. A phase II and phase III trial found several new ≥ 3 grade AEs, including dermal toxicity, hypertriglyceridemia, hyponatremia, and neutrophil count reduction, which were not reported in previous clinical trials [31, 44]. Thus, with the increasing number of anlotinib studies, potential long-term toxicity should be clarified.

Last, we know nearly nothing about tumor resistance to anlotinib and its possible mechanisms, as studies of anlotinib were just started in recent years. However, the knowledge of how to assess and reverse resistance to anlotinib is an important issue. Future trials should also develop personalized therapy strategies to conquer resistance.

## Conclusions

As a novel multi-targeting RTK inhibitor, anlotinib shows substantial antitumor activity against VEGFR1, VEGFR2/KDR, and VEGFR3, and lesser activity against c-kit, PDGFRα, FGFR1, FGFR2, and FGFR3. It is the first drug approved in China as a third-line treatment for patients with advanced NSCLC. With the development of future studies and accumulation of clinical experience, it is hopeful that anlotinib will be used in the treatment of other cancers, especially STS. In addition, anlotinib is well-tolerated and most AEs are manageable or reversible by medical intervention. Anlotinib has fewer or milder side effects compared to those of other anti-VEGFR TKIs, particularly the thrombocytopenia and neutropenia found with sunitinib. Thus, anlotinib is likely to become a new multi-targeting RTK inhibitor that is efficacious against multiple cancers.

## Abbreviations

aCECs: activated circulating endothelial cells
AE: Adverse event
ASPS: Alveolar soft part sarcoma
CFDA: China Food and Drug Administration
CI: Confidence interval
DCR: Disease control rate
EGFR: Epidermal growth factor receptor
FGFR: Fibroblast growth factor receptor
HR: Hazard ratio
mRCC: metastatic renal cell carcinoma
MTC: Medullary thyroid cancer
NSCLC: Non-small-cell lung cancer
ORR: Overall response rate
OS: Overall survival
PDGFR: Platelet-derived growth factor receptors
PFS: Progression-free survival
RTK: Receptor tyrosine kinase
STS: Soft tissue sarcoma
TKI: Tyrosine kinase inhibitor
VEGFR: Vascular endothelial growth factor receptor
